# Epidemiology and Risk Factors for HCV Infection Among MSM With or at Risk of HIV in Madrid (2022–2024)

**DOI:** 10.1093/ofid/ofaf678

**Published:** 2025-11-06

**Authors:** Pablo Ryan, Juan Berenguer, Luis Ramos-Ruperto, Mar Vera, Luz Martín-Carbonero, Leire Pérez-Latorre, Ignacio De los Santos, Adriana Pinto, María J Vivancos, Eva Orviz, Beatriz Álvarez, José Sanz, Pilar Ruiz-Seco, Rafael Torres, Beatriz Brazal, Marta De Miguel, Beatriz López-Centeno, Inmaculada Jarrín, Salvador Resino, José M Bellón, Juan González-García

**Affiliations:** Enfermedades Infecciosas, Hospital Universitario Infanta Leonor, Madrid, Spain; Instituto de Investigación Sanitaria Gregorio Marañón (IiSGM), Madrid, Spain; Centro de Investigación Biomédica en Red de Enfermedades Infecciosas (CIBERINFEC), Madrid, Spain; Universidad Complutense de Madrid (UCM), Madrid, Spain; Instituto de Investigación Sanitaria Gregorio Marañón (IiSGM), Madrid, Spain; Centro de Investigación Biomédica en Red de Enfermedades Infecciosas (CIBERINFEC), Madrid, Spain; Unidad de Enfermedades Infecciosas, Hospital General Universitario Gregorio Marañón, Madrid, Spain; Centro de Investigación Biomédica en Red de Enfermedades Infecciosas (CIBERINFEC), Madrid, Spain; Unidad de VIH/Servicio de Medicina Interna, Hospital Universitario La Paz, Madrid, Spain; Instituto de Investigación Sanitaria Hospital La Paz (IdiPAZ), Madrid, Spain; Centro de Investigación Biomédica en Red de Enfermedades Infecciosas (CIBERINFEC), Madrid, Spain; Centro Sanitario Sandoval, Madrid, Spain; Enfermedades Infecciosas, Hospital Clínico San Carlos, Madrid, Spain; Instituto de Investigación Sanitaria del Hospital Clínico de San Carlos (IdISSC), Madrid, Spain; Centro de Investigación Biomédica en Red de Enfermedades Infecciosas (CIBERINFEC), Madrid, Spain; Unidad de VIH/Servicio de Medicina Interna, Hospital Universitario La Paz, Madrid, Spain; Instituto de Investigación Sanitaria Hospital La Paz (IdiPAZ), Madrid, Spain; Instituto de Investigación Sanitaria Gregorio Marañón (IiSGM), Madrid, Spain; Centro de Investigación Biomédica en Red de Enfermedades Infecciosas (CIBERINFEC), Madrid, Spain; Unidad de Enfermedades Infecciosas, Hospital General Universitario Gregorio Marañón, Madrid, Spain; Centro de Investigación Biomédica en Red de Enfermedades Infecciosas (CIBERINFEC), Madrid, Spain; Enfermedades Infecciosas, Hospital Universitario de la Princesa, Madrid, Spain; Instituto de Investigación Sanitaria del Hospital de la Princesa (IIS-PRINCESA), Madrid, Spain; Centro de Investigación Biomédica en Red de Enfermedades Infecciosas (CIBERINFEC), Madrid, Spain; Unidad VIH, Hospital Universitario 12 de Octubre, Madrid, Spain; Instituto de Investigación Sanitaria Hospital 12 de Octubre (I+12), Madrid, Spain; Centro de Investigación Biomédica en Red de Enfermedades Infecciosas (CIBERINFEC), Madrid, Spain; Enfermedades Infecciosas, Hospital Universitario Ramón y Cajal, Madrid, Spain; Instituto Ramón y Cajal de Investigación Sanitaria (IRYCIS), Madrid, Spain; Centro de Investigación Biomédica en Red de Enfermedades Infecciosas (CIBERINFEC), Madrid, Spain; Enfermedades Infecciosas, Hospital Clínico San Carlos, Madrid, Spain; Instituto de Investigación Sanitaria del Hospital Clínico de San Carlos (IdISSC), Madrid, Spain; Enfermedades Infecciosas, Hospital Universitario Fundación Jiménez Díaz, Madrid, Spain; Enfermedades Infecciosas, Hospital Universitario Príncipe de Asturias, Alcalá de Henares, Spain; Medicina Interna, Hospital Universitario Infanta Sofía (FIIB HUIS-HUHEN), San Sebastián de los Reyes, Spain; Universidad Europea de Madrid, Madrid, Spain; Enfermedades Infecciosas, Hospital Universitario Severo Ochoa, Leganés, Spain; Fundación SEIMC-GeSIDA, Madrid, Spain; Fundación SEIMC-GeSIDA, Madrid, Spain; Subdirección General de Farmacia y Productos Sanitarios, Servicio Madrileño de Salud (SERMAS), Madrid, Spain; Centro de Investigación Biomédica en Red de Enfermedades Infecciosas (CIBERINFEC), Madrid, Spain; Centro Nacional de Epidemiología—Instituto de Salud Carlos III (ISCIII), Madrid, Spain; Centro de Investigación Biomédica en Red de Enfermedades Infecciosas (CIBERINFEC), Madrid, Spain; Centro Nacional de Microbiología—Instituto de Salud Carlos III (ISCIII), Majadahonda, Spain; Instituto de Investigación Sanitaria Gregorio Marañón (IiSGM), Madrid, Spain; Centro de Investigación Biomédica en Red de Enfermedades Infecciosas (CIBERINFEC), Madrid, Spain; Unidad de Enfermedades Infecciosas, Hospital General Universitario Gregorio Marañón, Madrid, Spain; Centro de Investigación Biomédica en Red de Enfermedades Infecciosas (CIBERINFEC), Madrid, Spain; Unidad de VIH/Servicio de Medicina Interna, Hospital Universitario La Paz, Madrid, Spain; Instituto de Investigación Sanitaria Hospital La Paz (IdiPAZ), Madrid, Spain

**Keywords:** acute HCV infection, DAA therapy, HCV, HCV reinfection, MSM

## Abstract

**Background:**

Ongoing high-risk behaviors continue to fuel HCV transmission among men who have sex with men (MSM), challenging elimination efforts. We studied HCV epidemiology in MSM with HIV (MSM-WH) and without HIV in the region of Madrid.

**Methods:**

This prospective study (2022–2024) enrolled MSM-WH from 10 centers and MSM on PrEP from an STI clinic. Visits were scheduled at baseline, 3, 6, 9, and 12 months (PrEP group), or baseline, 6, and 12 months (HIV group). Assessments included liver enzymes, HCV serology, HCV-RNA, and STI screening (syphilis, chlamydia, and gonorrhea by PCR).

**Results:**

A total of 1372 MSM (733 with HIV; 639 on PrEP) were enrolled. Baseline HCV prevalence was 1.68%, significantly higher in those with prior HCV exposure (5.60% vs 0.72%; prevalence ratio: 7.72, 95% CI: 3.31–18.03). Over 1240.4 person-years (PY) of follow-up, overall HCV incidence was 1.45/100 PY. Primary infection incidence was 0.79/100 PY: 0.94 in PrEP users versus 0.65 in MSM-WH (IRR: 1.44, 95% CI: .24–9.80). Reinfection incidence was 4.32/100 PY overall: 12.90 in PrEP users and 4.05 in MSM-WH (IRR: 3.21, 95% CI: .07–22.53). Two participants experienced within study reinfection (8.7/100 PY, 95% CI: 1.05–31.4). Slamsex and condomless receptive anal intercourse with ≥4 partners were independently associated with HCV infection and reinfection.

**Conclusions:**

MSM with prior HCV exposure had markedly higher HCV prevalence and incidence, regardless of HIV status. Risky sexual behaviors remain key drivers of HCV transmission. Behavior-informed prevention strategies are critical to sustain elimination efforts in MSM populations.

## Teaser

High-risk behaviors including slamsex and condomless receptive anal intercourse with multiple partners drive HCV transmission among MSM, irrespective of HIV status. Reinfection is common. Awareness, harm reduction, and targeted test-and-treat strategies are essential for elimination.

## Graphical Abstract

**Graphical Abstract:**
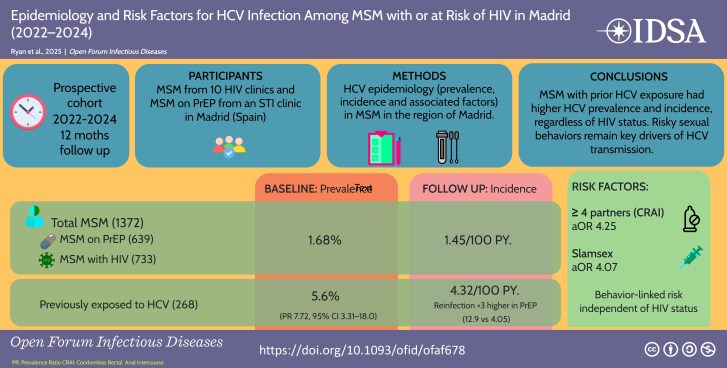
This graphical abstract is also available at Tidbit: https://tidbitapp.io/tidbits/epidemiology-and-risk-factors-for-hcv-infection-among-msm-with-or-at-risk-of-hiv-in-madrid-2022-2024-a38dff7d-0fbe-4c45-9b05-5bc0dd107a87?utm_campaign=tidbitlinkshare&utm_source=ITP

Historically, the spread of what was once termed “non-A, non-B hepatitis” was mainly linked to blood transfusions, unsafe medical practices, and shared injection drug equipment [1]. The identification of the hepatitis C virus (HCV) in 1989, followed by rigorous blood donor screening and improved healthcare protocols in the 1990s, led to a major decline in medically related transmissions and reshaped the disease's global epidemiology [2, 3].

Despite progress from screening and harm-reduction initiatives, injection drug use (IDU) remains the leading mode of HCV transmission worldwide [4]. In recent decades, high-risk behaviors among men who have sex with men (MSM) have become a significant concern, now the second most common route of transmission in many regions [5, 6]. These behaviors include condomless receptive anal intercourse (CRAI) with multiple partners and drug use in sexual contexts (chemsex), particularly when injected (slamsex), both of which heighten the risk of HCV acquisition [6]. Initially, new infections and reinfections occurred mainly among MSM with HIV-1 (HIV thereafter) engaged in these practices. More recently, however, rising cases have been reported among HIV-negative MSM, particularly those using pre-exposure prophylaxis (PrEP) [7, 8].

The advent of direct-acting antivirals (DAAs) has revolutionized HCV treatment, with cure rates exceeding 95% [9]. This advance is central to the World Health Organization's goal of eliminating viral hepatitis, including HCV, as a public health threat by 2030 [10]. Although global scale-up of DAA therapy has reduced HCV-related morbidity and mortality [11, 12], sustaining reductions in new infections remains challenging without an effective vaccine [13]. Recent studies report persistent HCV infections and reinfections among MSM engaging in high-risk behaviors, even in high-resource settings with wide DAA access [14–16]. These findings highlight the need for ongoing epidemiological monitoring, especially as treatment expansion has been linked to reductions in primary infections and reinfections [17, 18].

Given the evolving dynamics of HCV transmission, understanding local patterns and risk factors is essential for guiding targeted elimination strategies. We investigated HCV infections and reinfections among MSM with or at risk for HIV in the Madrid region. By focusing on this high-priority population, our aim is to inform elimination efforts by contributing data from a setting with broad access to DAAs.

## METHODS

### Study Design and Participants

This prospective cohort study enrolled MSM aged ≥18 years, either with HIV or using PrEP for HIV prevention, in the Madrid Region (Comunidad de Madrid), one of Spain's 17 autonomous communities, with approximately 7 million inhabitants.

MSM with HIV included in the Cohort of the Spanish Network of AIDS Research (CoRIS) since 2015 at 10 hospitals in the Madrid Region were eligible for participate. CoRIS is a national prospective cohort of antiretroviral therapy (ART)-naïve individuals with HIV established in 2004 [19]. To specifically address HCV reinfection, MSM with HIV who had cleared prior HCV infection after DAA therapy were selected from the Madrid Coinfection Registry (Madrid-CoRE), a mandatory registry covering people treated with DAAs across hospitals within the Madrilenian Health Service (SERMAS) since 2014 [20]. Individuals present in both databases were assigned to Madrid-CoRE. MSM on PrEP were recruited from Centro Sanitario Sandoval, a public sexually transmitted infections (STI) clinic in Madrid [21].

All data were pseudonymized prior to analysis. Visit schedules over a 12-month period followed pre-existing cohort/clinic protocols. MSM with HIV attended follow-ups at 6 (±2) and 12 (±2) months post-enrollment, whereas MSM on PrEP had quarterly visits according to the Madrid PrEP protocol. Enrollment ran from 1 May 2022, to 28 February 2024.

### Investigations

Baseline data for MSM with HIV were extracted from the CoRIS and Madrid-CoRE databases and supplemented with study-specific fields collected using electronic case report forms in REDCap [22] hosted by the SEIMC/GeSIDA Foundation, as detailed in Supplementary Material Appendix 1. For MSM on PrEP, baseline information came from the Centralized Registry of the Subdirectorate General for Pharmacy and Medical Devices, the official PrEP monitoring system in Madrid.

Variables collected included demographics, and HCV/STI history. For MSM with HIV, additional data included prior AIDS events and ART history. Participants underwent blood counts and liver enzyme tests at baseline and follow-up. HCV screening used serologic assays with confirmatory HCV-RNA testing or direct RNA testing in those with prior HCV. Gonorrhea and chlamydia screening was done via PCR on pharyngeal, urethral (or urine), and rectal samples. Syphilis screening used automated treponemal immunoassays, confirmed by quantitative non-treponemal tests. HIV serology was performed in MSM on PrEP; MSM with HIV had CD4 and HIV-RNA measured.

Behavioral data on sexual practices and drug use were collected longitudinally for MSM with HIV through a mobile app, capturing behavior in the prior 2 months before each visit (Supplementary Material Appendix 2). For MSM on PrEP, behavioral data were recorded only at PrEP initiation, covering the prior two months (Supplementary Material Appendix 3).

### Definitions and Outcomes

Prior HCV exposure was defined as any evidence of previous infection, including documented HCV-RNA positivity or anti-HCV positivity at baseline (regardless of RNA status). Primary HCV infection was defined as a PCR-confirmed infection in individuals without prior HCV exposure Reinfection was defined as a new HCV-RNA–positive result following documented clearance of a prior infection, either through spontaneous clearance or SVR. Within-study reinfection was defined as reinfection, per these criteria, that occurred during the study follow-up.

Primary outcomes included the baseline prevalence of active HCV infection (detectable RNA at enrollment) and the incidence rates of primary infection and reinfection, expressed per 100 person-years (PY). Person-time accrued from baseline and was censored at the first positive RNA test, the last available test, or end of follow-up, whichever occurred first. Within-study reinfections were analyzed separately. For these, person-time began at the first negative RNA test following a positive and was censored using the same criteria.

### Statistical Analysis

Descriptive statistics summarized baseline characteristics. Prevalence and incidence rates were calculated along with 95% confidence intervals (CIs), stratified by HIV status and prior HCV exposure. Participants with active HCV at baseline were excluded from incidence analyses. Prevalence ratios and incidence rate ratios (IRRs) with 95% CIs were estimated using Poisson regression to compare groups.

Factors associated with HCV acquisition were analyzed by grouping incident infections and recent prevalent infections, defined as infections estimated to have occurred within the previous 12 months based on the European Treatment Network for HIV, Hepatitis and Global Infectious Diseases (NEAT-ID) Consensus Panel criteria [6]. This grouping was justified by temporal proximity and similar characteristics, assuming comparable transmission dynamics.

The primary analysis, restricted to MSM with HIV due to the availability of detailed behavioral data, used logistic regression. Independent variables included age, country of birth, prior HCV infection, sexual behaviors, drug use, and concurrent STIs.

Behavioral variables were assessed categorically and ordinally, using the highest frequency reported across any visit. CRAI partner counts were dichotomized according to optimal cutoffs derived from receiver operating characteristic (ROC) analysis and the Youden index. STIs were classified as positive if diagnosed at baseline or during follow-up. Variables with *P* < .05 in univariate analysis were included in multivariable models. Temporal trends in behaviors were tested via chi-squared and independent-group tests; within-subject behavioral changes were analyzed using Stuart-Maxwell and marginal homogeneity tests.

Sensitivity analyses included generalized estimating equations (GEE) to account for repeated measures among MSM with HIV; separate logistic and GEE models to distinguish primary infections from reinfections; and Firth's penalized logistic regression applied to the full cohort regardless of HIV status, using common baseline variables to mitigate sparse-event bias.

Missing data were handled using multiple imputation by chained equations. Little's test was used to evaluate the assumption of missing completely at random.

All tests were two-sided, and analyses were performed using SPSS v25 and STATA v18.0. Study reporting adhered to STROBE guidelines (Supplementary Table 1), with further methodological details available in Supplementary Material Appendix IV.

## ETHICAL CONSIDERATIONS

The study was conducted in accordance with the Declarations of Helsinki and Istanbul and received approval from the Ethics Committee of Hospital General Universitario Gregorio Marañón (Study Code GESIDA 12151, Minutes 14/2021, dated 5 July 2021). Written informed consent was obtained from all participants, and all procedures were compliant with local regulations on data confidentiality.

## RESULTS

### Participants

Of 2109 eligible MSM with HIV, 733 consented and were enrolled; 1376 were excluded due to lack of consent request, refusal, relocation, or loss to follow-up. Baseline differences between enrolled and non-enrolled participants are detailed in Supplementary Table 2. In CoRIS, enrolled MSM were more often native-born Spaniards (58.3% vs 47.5%); in Madrid-CoRE, more were on ART (99.6% vs 97.0%). No other statistically significant differences emerged.

Baseline characteristics of the 1372 participants (733 MSM with HIV and 639 MSM on PrEP) are summarized in Table 1. One individual included in both cohorts was assigned to Madrid-CoRE, as per the predefined criteria. MSM with HIV were older (41 vs 37 years) and more often native-born Spaniards. High rates of CRAI (65.6% vs 97.5%) and previous STIs (75.0% vs 67.8%) were reported by both groups. Chemsex was reported by 33.3% of MSM with HIV and 27.0% MSM on PrEP. Nearly all MSM with HIV (99.6%) were on ART, 94.4% had undetectable HIV viral loads, and the median CD4+ count was 777 cells/µL.

**Table 1.** Baseline Characteristics of 1372 Study Participants According to Their Cohort of Origin

Characteristic	MSM With HIV CoRIS	MSM With HIV Madrid-CoRE	All MSM With HIV	MSM Without HIV Madrid-PrEP	*P* ^a^
	*N* = 492	*N* = 241	*N* = 733	*N* = 639	
Demographics					
Age, years, median (IQR)	38 (31–45)	48/241 (41–55)	41/733 (34–49)	37 (33–44)	<.001
Born in Spain, *n*/*N* (%)	287/492 (58.3)	160/241 (66.4)	447/733 (61.0)	368 (57.6)	.205
Born in Latin America/Caribbean, *n*/*N* (%)	183/492 (37.2)	72/241 (29.9)	255/733 (34.8)	NA	
Born in other countries, *n*/*N* (%)	22/492 (4.5)	9/241 (3.7)	31/733 (4.2)	NA	
HIV-related variables					
Prior AIDS defining categories, *n*/*N* (%)	49/492 (10.0)	35/241 (14.5)	84/733 (11.5)	NA	
On ART, *n*/*N* (%)	489/492 (99.4)	241/241 (100.0)	730/733 (99.6)	NA	
CD4 cell count, median (IQR)	786 (596–983)	773 (555–973)	777 (591–979)	NA	
HIV RNA < 50 copies/mL, *n*/*N* (%)	464/492 (94.3)	228/241 (94.6)	692/733 (94.4)	NA	
Risk practices for STI/HCV					
CRAI in the previous 2 m, *n*/*N* (%)	229/353 (64.9)	124/185 (67.0)	353/538 (65.6)	623/639 (97.5)	<.001
Chemsex, *n*/*N* (%)	90/354 (25.4)	90/186 (48.4)	180/540 (33.3)	157/582 (27.0)	.023
Slamsex, *n*/*N* (%)	12/354 (3.4)	29/186 (15.6)	41/540 (7.6)	18/361 (5.0)	.132
Prior history of HCV/STI					
HCV infection, *n*/*N* (%)	13/492 (2.6)	241/241 (100)	254/733 (34.7)	14/515 (2.7)	<.001
Any STI, *n*/*N* (%)	332/492 (67.5)	218/241 (90.5)	550/733 (75.0)	433/639 (67.8)	.003
Syphilis, *n*/*N* (%)	286/491 (58.2)	208/241 (86.3)	494/732 (67.5)	106/414 (25.6)	<.001
*Neisseria gonorrhoeae*, *n*/*N* (%)	146/443 (33.0)	89/205 (43.4)	235/648 (36.3)	134/414 (32.4)	.209
*Chlamydia trachomatis*, *n*/*N* (%)	95/438 (21.7)	62/200 (31.0)	157/638 (24.6)	135/414 (32.6)	.005

*n*/*N* (%) indicates the number of participants with the characteristic over the number assessed (%).

Abbreviations: ART, antiretroviral therapy; Chemsex, drug use during sex by any route; CRAI, condomless receptive anal intercourse; IQR, interquartile range; MSM, men who have sex with men; NA, not available; Slamsex, injection drug use during sex; STI, sexually transmitted infection.

^a^Refers to comparisons between all MSM with HIV and MSM without HIV.

### HCV Infections

A total of 41 HCV infections were identified in 39 participants: 30 in 29 MSM with HIV, and 11 in 10 MSM on PrEP. Table 2 shows HCV prevalence and incidence stratified by prior HCV exposure and HIV status.

**Table 2.** Prevalence and Incidence of HCV Infection Categorized by Prior HCV Exposure and HIV Status

Participant Category	No	No With Prevalent HCV	HCV Prevalence % (95% CI)	Years FU	No With Incident HCV	HCV Incidence Per 100 PY (95% CI)
All MSM	1372	23	1.68 (1.07–2.50)	1240.4	18	1.45 (.91–2.30)
No prior HCV exposure	1104	8	0.72 (.31–1.42)	884.3	7	0.79 (.38–1.66)
MSM without HIV	625	3	0.48 (.10–1.40)	425.8	4	0.94 (.35–2.50)
MSM with HIV	479	5	1.04 (.34–2.42)	458.6	3	0.65 (.21–2.03)
Prior HCV exposure	268	15	5.60 (3.17–9.06)	254.5	11	4.32 (2.391–7.80)
MSM without HIV	14	3	21.4 (4.66–50.8)	7.7	1	12.92 (1.82–91.7)
MSM with HIV	254	12	4.72 (2.46–8.11)	246.8	10	4.05 (2.18–7.53)

Abbreviations: CI, confidence interval; FU, follow-up; HCV, Hepatitis C virus; HIV, Human Immunodeficiency Virus; MSM, men who have sex with men; PY, Person-years.

Baseline HCV prevalence was 1.68%, higher in those with prior HCV exposure (5.60%) versus those without (0.72%; prevalence ratio: 7.72, 95% CI: 3.31–18.03).

During 1240.4 PY of follow-up, overall HCV incidence was 1.45 per 100 PY. Among those without prior HCV exposure, primary infection incidence was 0.79 per 100 PY overall: 0.94 per 100 PY among MSM on PrEP, and 0.65 per 100 PY among MSM with HIV (IRR: 1.44; 95% CI: .24–9.80). Among those with prior HCV exposure, reinfection incidence was 4.32 per 100 PY overall: 12.92 per 100 PY among MSM on PrEP, and 4.05 among MSM with HIV (IRR: 3.21; 95% CI: .07–22.53).

Two within-study reinfections occurred (one in each group) yielding a within-study reinfection rate of 8.7 per 100 PY (95% CI: 1.05–31.4).

Symptom data were available for 40 episodes, of which 5 (12.5%) were symptomatic. Of the 41 infections, 3 resolved spontaneously, and in 3 the participants were lost to follow-up after diagnosis. Among the 35 infections treated with DAAs, 34 achieved SVR and one participant was lost to follow-up before the treatment response could be assessed.

### Risk Factors for HCV Infections

To assess potential selection bias within the subgroup of MSM with HIV included in the primary analysis, we compared baseline characteristics and HCV prevalence between those who completed at least one behavioral questionnaire (*N* = 540) and those who did not (*N* = 193). No clinically relevant differences were observed (Supplementary Table 3).

Risk behaviors among MSM with HIV from baseline to month 12, assessed by independent and repeated measures analyses, are summarized in Supplementary Tables 4 and 5. Overall, sexual behaviors remained stable over time; however, some drug-related variables—particularly mephedrone consumption and engagement in chemsex—showed modest yet statistically significant reductions. The optimal cutoff for the number of CRAI partners, identified through ROC analysis and the Youden index, was ≥4. This threshold was consistent whether considering baseline behavioral questionnaires (one per participant) or all questionnaires across visits (up to three observations per participant) (Supplementary Table 6A and B).

The primary analysis (Table 3) included 540 MSM with HIV with at least one questionnaire, and 21 HCV infections. Bivariate analyses associated prior HCV infection, CRAI, CRAI with ≥4 partners, fisting, drug use frequency, chemsex, slamsex, needle sharing, and specific substances with HCV infection. Age, country of birth, and concurrent STIs showed no significant associations. Multivariate analysis confirmed CRAI with ≥4 partners (aOR: 4.25; 95% CI: 1.56–11.60) and slamsex (aOR: 4.07; 95% CI: 1.10–15.05) as independent predictors. Sensitivity analyses using GEE identified slamsex as the sole independent risk factor (aOR: 3.50; 95% CI: 1.29–9.50) (Supplementary Table 7).

**Table 3.** **Logistic Regression Analysis of Variables Associated With HCV Infection Events Among MSM With HIV** ^a^

Variable^b^	Univariate OR (95% CI)	*P*	Multivariate OR (95% CI)	*P*
Age	1.01 (.97–1.05)	.715	-	-
Born in Spain	0.99 (.40–2.44)	.990	-	-
History of prior HCV	3.73 (1.48–9.43)	.005	1.85 (.63–5.38)	.260
CRAI	11.08 (1.47–83.36)	.020	3.76 (.45–31.48)	.222
CRAI ≥ 4 partners	12.85 (4.24–38.92)	<.001	4.25 (1.56–11.60)	.005
Fisting	6.35 (2.59–15.55)	<.001	1.48 (.56–3.93)	.434
STI any	2.13 (.88–5.18)	.094	-	-
Syphilis	1.70 (.55–5.23)	.325	-	-
Gonorrhea	2.62 (.80–6.39)	.124	-	-
Chlamydia	0.44 (.06–3.35)	.428	-	-
Frequency of drug use	0.64 (.49–.84)	.001	.73 (.46–1.78)	.199
Chemsex	11.20 (3.25–38.59)	<.001	.96 (.13–7.01)	.966
Methamphetamine use	9.83 (3.98–24.29)	<.001	1.94 (.69–5.41)	.208
Mephedrone use	7.48 (2.84–19.70)	<.001	.52 (.12–2.15)	.367
GHB use	8.38 (3.29–21.32)	<.001	1.66 (.51–5.45)	.401
Needle Sharing	7.70 (1.50–39.63)	.015	.76 (.11–5.13)	.774
Slamsex	11.19 (4.47–28.00)	<.001	4.07 (1.10–15.05)	.036

Abbreviations: Chemsex, drug use during sex; CI, confidence interval; CRAI, condomless receptive anal intercourse; GHB, gamma-hydroxybutyrate; HCV, hepatitis C virus; OR, odds ratio; Slamsex, intravenous drug use during sex; STI, sexually transmitted infection.

^a^HCV infection events include both primary infections and reinfections (*n* = 21) identified among 540 MSM with HIV who completed at least one app-based questionnaire.

^b^Variables related to sexual activity and drug use reflect behaviors in the two months preceding questionnaire completion. The threshold of ≥4 CRAI partners during this two-month period was determined using ROC curves and the Youden index. STIs refer to any diagnosis of a sexually transmitted infection during the study period. The frequency of drug use was assessed as an ordinal variable with the following categories: daily, weekly, biweekly, monthly, annually, or no drug use.

Only two behavioral variables—CRAI with ≥4 partners and slamsex—were included in stratified models by prior HCV exposure status, as these were the only factors significantly associated with HCV infections in the primary analysis. Among those without prior HCV exposure, both were significantly associated with HCV infection in logistic regression and GEE models. Among those with prior exposure, CRAI ≥4 partners was significantly associated with reinfection in the logistic model (OR, 6.14; 95% CI, 1.61–23.44), while slamsex did not reach significance (OR, 2.78; 95% CI, .85–9.08). However, slamsex was significantly associated with reinfection in the GEE model (OR, 3.91; 95% CI, 1.24–12.33), and CRAI ≥4 partners showed a trend toward significance (OR, 3.18; 95% CI, .96–10.46). Full results are presented in Table 4.

**Table 4.** Stratified Analysis by Prior HCV Exposure Status Among MSM With HIV

Analysis	Variable	No Prior HCV Exposure^a^ Multivariate OR (95% CI)	*P*	Prior HCV Exposure^b^ Multivariate OR (95% CI)	*P*
Primary^c^	CRAI partners ≥ 4	11.38 (1.18–109.53)	.035	6.14 (1.61–23.44)	.008
	Slamsex	15.10 (2.68–82.25)	.002	2.78 (.85–9.08)	.090
Secondary^d^	CRAI partners ≥ 4	7.29 (1.35–39.32)	.021	3.18 (.96–10.46)	.057
	Slamsex	11.17 (2.82–44.26)	.001	3.91 (1.24–12.33)	.020

HCV infection events include both primary infections and reinfections (*n* = 21), identified among 540 MSM with HIV who completed at least one app-based questionnaire. Only CRAI with ≥4 partners and slamsex were included in stratified models, as these were the only variables associated with HCV infections in the primary analysis.

Abbreviations: Chemsex, drug use during sex; CI, confidence interval; CRAI, condomless receptive anal intercourse; GHB, gamma-hydroxybutyrate; HCV, hepatitis C virus; OR, odds ratio; Slamsex, intravenous drug use during sex; STI, sexually transmitted infection.

^a^345 participants, 7 HCV events.

^b^195 participants, 14 HCV events.

^c^Logistic regression models: exposures (CRAI with ≥4 partners or slam sex) coded as positive if reported at any visit (baseline, month 6, or month 12).

^d^Generalized estimating equation (GEE) models accounting for within-subject correlation across time points.

Firth's penalized logistic regression (MSM with HIV and MSM on PrEP) identified slamsex (OR: 4.49) and prior HCV infection (OR: 2.74) as significant risk factors. Chemsex showed a borderline association, while CRAI showed a high odds ratio (OR: 13.93) but wide confidence intervals due to the absence of cases in the comparator group. Other factors (age, country of birth, HIV status, previous STIs) were not significantly associated (Supplementary Table 8).

## DISCUSSION

In this prospective cohort study conducted in Madrid between 2022 and 2024 among 1372 MSM, we observed a baseline active HCV infection prevalence of 1.68%, significantly higher among those previously exposed to HCV (5.60%) than those without exposure (0.72%). The incidence rates of primary HCV infection and reinfection were 0.79 and 4.32 per 100 person-years, respectively, with no significant differences between MSM with HIV and MSM on PrEP. Slamsex and CRAI with multiple partners emerged as consistent independent risk factors for both infection types.

Our findings align with meta-analytic estimates from other European and high-income regions, where active HCV prevalence among MSM with HIV is approximately 2.40% [23]. Among MSM on PrEP, our prevalence (0.94%) matches recent North American estimates (0.82%–0.84%) [24, 25], although it exceeds earlier pooled global estimates (0.38%) [8] reflecting substantial international variability.

The observed primary HCV incidence among MSM with HIV and MSM on PrEP (0.65–0.94 per 100 PY) are consistent with prior reports [8, 23], though international differences remain notable [26–28]. The observed reinfection rate among MSM with HIV (4.05 per 100 PY) matches rates reported in New York City [29], yet remains lower than in Germany (up to 15.5 per 100 PY) [30, 31]. Compared with Madrid's initial DAA-era estimate (5.93 per 100 PY) [15], our data indicate a 32% decrease, suggesting progress in controlling reinfection or a treatment-as-prevention effect, yet highlighting an ongoing concern. Among MSM on PrEP, the reinfection rate (12.92 per 100 PY) falls within the previously reported international range [26, 27].

Although HCV prevalence and incidence were numerically higher among MSM on PrEP, these differences were not statistically significant, contrasting with earlier studies reporting higher rates in this group [23, 26, 28], This aligns with phylogenetic evidence of overlapping transmission networks among MSM regardless of HIV status [26, 32, 33] supporting unified prevention strategies.

Slamsex and CRAI with multiple partners were independently associated with HCV acquisition among MSM with HIV in our primary analysis. Notably, having four or more partners marked a risk threshold. This analysis was based on app-based questionnaires assessing both primary infections and reinfections. A sensitivity analysis using GEE in this subgroup identified slamsex as the sole significant risk factor. Another sensitivity analysis including the full MSM cohort, irrespective of HIV status, confirmed slamsex and prior HCV infection as significant predictors. CRAI showed a high odds ratio, but wide confidence intervals limited its interpretability due to the absence of cases in the comparator group. These results support prior evidence identifying slamsex and CRAI with multiple partners as key drivers of HCV transmission among MSM [32–35]. However, concurrent STIs, commonly described as biological cofactors [26, 36], were not significantly associated with HCV infection in our cohort.

Stratified analyses revealed overlapping and model-dependent risk patterns. Among those without prior HCV, both slamsex and CRAI with ≥4 partners were significantly associated with primary infection across analytical approaches, including logistic regression and GEE. Among those with prior HCV, results varied: CRAI with ≥4 partners was associated with reinfection only in logistic regression, whereas slamsex reached significance only in the GEE model. The logistic regression estimate for slamsex, although not statistically significant (*P* = .090), suggested a potentially meaningful association with wide confidence intervals. Similarly, CRAI ≥4 partners had a borderline association in the GEE model (*P* = .057). These discrepancies likely reflect how each method handles repeated measures and intra-individual correlation and may also be influenced by the number of reinfections. Rather than dismissing these findings, the direction and strength of associations support the relevance of both risk factors, possibly exerting time-dependent or context-specific effects.

In contrast, Fierer et al. reported a more distinct behavioral dichotomy between primary infections and reinfections, linking the latter primarily to CRAI involving rectal semen exposure while attributing primary infections to methamphetamine use, regardless of route [29]. These differences suggest that associations between specific behaviors and reinfection may vary depending on methodological approaches, highlighting the complexity of reinfection dynamics and underscoring potential regional or cohort-specific factors influencing HCV transmission patterns.

Several limitations should be considered. First, the generalizability of our findings may be limited by the specific study population and setting. Second, the one-year follow-up period may not fully capture long-term transmission trends. Third, behavioral data were collected exclusively among MSM with HIV due to the study design, and questionnaire completion was not universal. However, no clinically relevant differences were observed between participants who completed at least one questionnaire and those who did not, suggesting minimal risk of selection bias. Fourth, differences in visit frequency between groups could introduce modest detection bias. Nevertheless, incidence was calculated per person-time using RNA-confirmed outcomes, with event dates assigned within the corresponding intervals. Thus, while more frequent testing may shorten detection intervals, it is unlikely to artificially increase event counts. This approach minimizes—though does not fully exclude—potential bias. Finally, the relatively low number of primary infections constrained the power to detect more subtle epidemiological differences. Nonetheless, this study has several strengths, including its prospective design, large and contemporaneously recruited cohort during widespread DAA availability, use of robust laboratory diagnostics, detailed behavioral data for a large subset of participants, and parallel analysis of MSM with HIV and MSM on PrEP.

In conclusion, despite the widespread availability of DAAs, HCV remains a significant public health concern among MSM in Madrid, particularly among individuals engaging in high-risk practices such as slamsex and CRAI with multiple partners. These findings underscore the ongoing vulnerability of this population even in well-resourced settings with universal access to treatment. Spain is internationally regarded as a reference in HCV elimination [37, 38], with micro-elimination nearly achieved among PWH [39]. Nevertheless, ongoing transmission among MSM, as highlighted by our study, indicates that specific gaps remain and may represent a barrier to the final steps toward micro-elimination. Targeted interventions are needed and should include harm-reduction services, accessible HCV screening integrated within both HIV care and PrEP programs, expanded testing coverage, community-based education, and MSM-specific surveillance systems. Comprehensive approaches addressing both behavioral and biological drivers of transmission are essential to achieving HCV micro-elimination goals in this key population.

## Supplementary Material

ofaf678_Supplementary_Data

## Supplementary Data

Supplementary materials are available at *Open Forum Infectious Diseases* online. Consisting of data provided by the authors to benefit the reader, the posted materials are not copyedited and are the sole responsibility of the authors, so questions or comments should be addressed to the corresponding author.
