# Supplementary material for: Epidemiology and Risk Factors for HCV Infection Among MSM With or at Risk of HIV in Madrid (2022–2024)
Source: Open Forum Infect Dis. 2025 Nov 6;12(12):ofaf678. doi: 10.1093/ofid/ofaf678 (PMC12651544; doi:10.1093/ofid/ofaf678)
Supplement: ofaf678_Supplementary_Data [file ofaf678_supplementary_data.pdf]

ATHENS STUDY (Study Code: GeSIDA 12121-RIS EPICLIN 08_2021)

Acute/Recent Infec**T**ions and Reinfections by **H**CV In M**EN** Having **S**ex with Men with and Without HIV.

Table of Contents

Item	Pages
**Table S1**. STROBE Statement—Checklist of items that should be included in reports of cohort studies	2
**Table S2**. Baseline characteristics of HIV-positive MSM from CoRIS and Madrid-CoRE that were included and not included in the study.	3
**Table S3.** Baseline characteristics and HCV prevalence among MSM with HIV, by completion of at least one behavioral questionnaire	4
**Table S4**. Summary of changes in risk practices over three time points (independent groups).	5
**Table S5**. Summary of changes in risk practices repeated measures summary	6
**Table S6.** Diagnostic performance of dichotomized CRAI partner counts using optimal cut-offs derived from ROC analysis and the Youden index	7
**Table S7**. Variables associated with HCV infections among 540 MSM with HIV who completed at least one App-based questionnaire, using generalized estimating equation (GEE) models to account for within-subject correlations over time.	8
**Table S8**. Multivariate analysis of factors associated with HCV infections among MSM participants with or without HIV infection using Firth's penalized logistic regression with multiple imputation.	9
**Appendix I:** Baseline and Follow-up Assessments for MSM with HIV (eCRF-Based)	**10-11**
**Appendix II:** App-Based Data on Sexual Practices, Drug Use, and Mental Health in MSM with HIV	**11-12**
**Appendix III:** Baseline and Follow-up Assessments for MSM without HIV on PrEP	**13-15**
**Appendix IV:** Detailed Statistical Analysis	**16-17**
**Appendix V.** GeSIDA 12121-RIS EPICLIN 08_2021 ATHENS Study Group	**18**

Table S1. STROBE Statement—Checklist of items that should be included in reports of ***cohort studies***

	Item	Recommendation	Manuscript location
**Title and abstract**	1	(*a*) Indicate the study's design with a commonly used term in the title or the abstract	Title and abstract
		(*b*) Provide in the abstract an informative and balanced summary of what was done and what was found	Title and abstract
**Introduction**			
Background/rationale	2	Explain the scientific background and rationale for the investigation being reported	Introduction
Objectives	3	State specific objectives, including any prespecified hypotheses	Introduction
**Methods**			
Study design	4	Present key elements of study design early in the paper	Methods (study design and participants)
Setting	5	Describe the setting, locations, and relevant dates, including periods of recruitment, exposure, follow-up, and data collection	Methods (study design and participants)
Participants	6	(*a*) Give the eligibility criteria, and the sources and methods of selection of participants. Describe methods of follow-up	Methods (study design and participants)
		(*b*) For matched studies, give matching criteria and number of exposed and unexposed	Non applicable
Variables	7	Clearly define all outcomes, exposures, predictors, potential confounders, and effect modifiers. Give diagnostic criteria, if applicable	Methods (investigations, Outcomes)). Supplementary material Appendix 1 and 2.
Data sources/ measurement	8*	For each variable of interest, give sources of data and details of methods of assessment (measurement). Describe comparability of assessment methods if there is more than one group	Methods (investigations)
Bias	9	Describe any efforts to address potential sources of bias	Methods (study design and participants)
Study size	10	Explain how the study size was arrived at	Non applicable
Quantitative variables	11	Explain how quantitative variables were handled in the analyses. If applicable, describe which groupings were chosen and why	Methods (Statistical analysis)
Statistical methods	12	(*a*) Describe all statistical methods, including those used to control for confounding	Methods (Statistical analysis)
		(*b*) Describe any methods used to examine subgroups and interactions	Methods (Statistical analysis)
		(*c*) Explain how missing data were addressed	Methods (Statistical analysis)
		(*d*) If applicable, explain how loss to follow-up was addressed	Methods (Statistical analysis)
		(*e*) Describe any sensitivity analyses	Methods (Statistical analysis)
**Results**			
Participants	13*	(a) Report numbers of individuals at each stage of study—e.g. numbers potentially eligible, examined for eligibility, confirmed eligible, included in the study, completing follow-up, and analyzed	Results (participants)
		(b) Give reasons for non-participation at each stage	Results (participants)
		(c) Consider use of a flow diagram	FALTA
Descriptive data	14*	(a) Give characteristics of study participants (e.g. demographic, clinical, social) and information on exposures and potential confounders	Results (participants). Table 1.
		(b) Indicate number of participants with missing data for each variable of interest	FALTA
		(c) Summarize follow-up time (e.g., average and total amount)	Results (HCV infections), Table 2
Outcome data	15*	Report numbers of outcome events or summary measures over time	Results (HCV infections), Table 2
Main results	16	(*a*) Give unadjusted estimates and, if applicable, confounder-adjusted estimates and their precision (eg, 95% confidence interval). Make clear which confounders were adjusted for and why they were included	Results (HCV infections), Table 2
		(*b*) Report category boundaries when continuous variables were categorized	Non applicable
		(*c*) If relevant, consider translating estimates of relative risk into absolute risk for a meaningful time period	Non applicable
Other analyses	17	Report other analyses done—e.g. analyses of subgroups and interactions, and sensitivity analyses	Results (Risk factors for HCV infections). Supplementary material
**Discussion**			
Key results	18	Summarize key results with reference to study objectives	Discussion section
Limitations	19	Discuss limitations of the study, taking into account sources of potential bias or imprecision. Discuss both direction and magnitude of any potential bias	Discussion section
Interpretation	20	Give a cautious overall interpretation of results considering objectives, limitations, multiplicity of analyses, results from similar studies, and other relevant evidence	Discussion section
Generalizability	21	Discuss the generalizability (external validity) of the study results	Discussion section
**Other information**			
Funding	22	Give the source of funding and the role of the funders for the present study and, if applicable, for the original study on which the present article is based	Funding section

Note: An Explanation and Elaboration article discusses each checklist item and gives methodological background and published examples of transparent reporting. The STROBE checklist is best used in conjunction with this article (freely available on the Web sites of PLoS Medicine at http://www.plosmedicine.org/, Annals of Internal Medicine at http://www.annals.org/, and Epidemiology at http://www.epidem.com/). Information on the STROBE Initiative is available at http://www.strobe-statement.org.

Table S2. Baseline characteristics of HIV-positive MSM from CoRIS and Madrid-CoRE that were included and not included in the study.

	Not Included	Included	Total	P	Missing, n (%)
**CoRIS, n (%)**	**988 (66.8)**	**492 (33.2)**	**1480 (100.0)**		
– Age, median (IQR)	31.8 (26.5; 39)	32.5 (26.8; 39.7)	31.9 (26.6; 39.4)	0.432	72/1480 (4.9)
– Native born Spaniard, n (%)	468 (47.5)	287 (58.3)	755 (51.1)	<0.001	3/1480 (0.2)
– Prior AIDS, n (%)	55 (5.6)	29 (6.1)	84 (5.7)	0.720	13/1480 (0.9)
– CD4 cell count, median (IQR)	419 (274; 610)	430 (279; 580)	423 (275; 600)	0.966	82/1480 (5.5)
– Undetectable HIV RNA, n (%)	19 (2.0)	9 (1.9)	28 (2.0)	1.000	76/1480 (5.1)
**Madrid-CoRE, n (%)**	**388 (61.7)**	**241 (38.3)**	**629 (100.0)**		
– Age, median (IQR)	42 (34.9; 48.8)	43.2 (37.3; 51.1)	42.5 (36; 49.6)	0.089	4/629 (0.6)
– Prior AIDS, n (%)	60 (15.5)	34 (14.3)	94 (15.0)	0.730	4/629 (0.6)
– On ART, n (%)	360 (97.0)	226 (99.6)	586 (98.0)	0.036	31/629 (4.9)
– CD4 cell count, median (IQR)	662 (492; 909)	744 (513; 961)	703 (498; 935)	0.125	155/629 (24.6)
– Undetectable HIV RNA, n (%)	330 (90.9)	200 (92.6)	530 (91.5)	0.539	50/629 (7.9)

Table S3. Baseline characteristics and HCV prevalence among MSM with HIV, by completion of at least one behavioral questionnaire.

Variable	Completed ≥1 questionnaire (N = 540)	No questionnaire (N = 193)	*P*-value
Age, median (IQR), years	41 (33–49)	39 (34–48)	0.325
Native-born Spaniard, n/N (%)	335/540 (62.0)	112/193 (58.0)	0.345
Prior AIDS diagnosis, n/N (%)	61/533 (11.4)	23/192 (12.0)	0.895
On ART, n/N (%)	539/540 (99.8)	191/193 (99.0)	0.171
CD4 cell count, median (IQR), cells/µL	802 (608–998)	740 (555-939)	0.010
Undetectable HIV RNA, n/N (%)	510/540 (94.4)	182/193 (94.3)	1.000
History of HCV infection, n/N (%)	195/540 (36.1)	59/193 (30.6)	0.186
Any previous STI, n/N (%)	410/540 (75.9)	140/193 (72.5)	0.383
Prior syphilis, n/N (%)	367/540 (68.0)	127/193 (65.8)	0.592
Prior *Neisseria gonorrhoeae* infection, n/N (%)	167/540 (30.9)	68/193 (35.2)	0.282
Prior *Chlamydia trachomatis* infection, n/N (%)	123/540 (22.8)	34/193 (17.6)	0.152
Baseline HCV prevalence, % (95% CI)	2.22 (1.15–3.85)	2.59 (0.85–5.94)	0.782

Abbreviations: MSM, men who have sex with men; IQR, interquartile range; STI, sexually transmitted infection.

Table S4. Summary of changes in risk practices over three time points (independent groups).

Variable	Baseline	Month 6	Month 12	P-value
**Number of participants**	540	319	164	
**Number of CRAI partners; median (IQR)**	3 (1; 5)	2 (1; 5)	2 (1; 5.5)	0.465
**Drug use, n (%)**	239 (44.4)	134 (42.5)	66 (40.2)	0.625
**Mephedrone use, n (%)**	133 (24.6)	73 (22.9)	34 (20.7)	0.584
**Methamphetamine use, n (%)**	60 (11.1)	32 (10.0)	16 (9.8)	0.860
**GHB use, n (%)**	99 (18.3)	57 (17.9)	25 (15.2)	0.684
**Fisting, n (%)**	61 (11.4)	35 (11.0)	16 (9.8)	0.881
**Chemsex participation, n (%)**	180 (33.3)	95 (29.8)	50 (30.5)	0.519

P-values were calculated using tests for comparisons between independent groups: the Chi-squared test for categorical variables and the Mann-Whitney test for non-parametric continuous variables.

Abbreviations: CRAI, condomless receptive anal intercourse; IQR, interquartile range; GHB, Gamma-hydroxybutyrate.

Table S5. Summary of changes in risk practices repeated measures summary

Variable	Comparison	Total (N)	Yes Baseline (N, %)	Yes Follow-up (N, %)	Median (IQR)	P
Nº of CRAI Partners	Baseline vs. M6	149	-	-	3 (1; 5) → 3 (1; 5)	0.767
	Baseline vs. M12	71	-	-	2 (1; 6) → 2 (1; 6)	0.140
Drug Use	Baseline vs. M6	314	150 (48)	134 (43)	-	0.024
	Baseline vs. M12	164	77 (47)	66 (40)	-	0.022
Mephedrone	Baseline vs. M6	319	90 (28)	73 (23)	-	0.005
	Baseline vs. M12	164	44 (27)	34 (21)	-	0.033
Methamphetamine	Baseline vs. M6	319	36 (11)	32 (10)	-	0.433
	Baseline vs. M12	164	20 (12)	16 (10)	-	0.317
GHB	Baseline vs. M6	319	61 (19)	57 (18)	-	0.480
	Baseline vs. M12	164	32 (20)	25 (15)	-	0.071
Fisting	Baseline vs. M6	317	38 (12)	35 (11)	-	0.564
	Baseline vs. M12	163	18 (11)	16 (10)	-	0.593
Chemsex	Baseline vs. M6	319	116 (36)	95 (30)	-	0.004
	Baseline vs. M12	164	62 (38)	50 (31)	-	0.011

P-value indicates statistical significance of changes over time. They were calculated using statistical tests tailored to the data type and comparison context. For continuous variables, Wilcoxon signed-rank test were likely used due to the non-normal distribution of medians and interquartile ranges. Binary variables were analyzed with McNemar's test to evaluate changes in paired proportions over time. Multinomial categorical variables (e.g., chemsex) were assessed using tests like the Stuart-Maxwell or Cochran's Q test, which handle repeated measures with multiple categories. General comparisons between groups were performed using symmetry and marginal homogeneity tests (e.g. Stuart-Maxwell) for repeated measures. These robust methods, which include tests for symmetry and marginal homogeneity, are suitable for analyzing behavioral changes or intervention effects in longitudinal studies with categorical or ordinal data.

Abbreviations: CRAI, condomless receptive anal intercourse; IQR, interquartile range; GHB, Gamma-hydroxybutyrate.

Table S6. Diagnostic performance of dichotomized CRAI partner counts using optimal cut-offs derived from ROC analysis and the Youden index among MSM with HIV.

A. Considering participants who completed the behavioral questionnaire at baseline. (N = 540; 21 events).

Cut-off	SEN % (95% CI)	SPE % (95% CI)	PPV % (95% CI)	NPV % (95% CI)	Youden Index	*P*
≥ 1	95.2 (76.2-99.9)	43.7 (39.4-48.1)	6.4 (4.0-9.7)	99.6 (97.6-100.0)	39.0	<0.001
≥ 2	85.7 (63.7-97.0)	62.0 (57.7-66.2)	8.4 (5.0-12.9)	99.1 (97.3-99.8)	47.8	<0.001
≥ 3	76.2 (52.8-91.8)	72.6 (68.6-76.4)	10.1 (5.9-15.9)	98.7 (97.0-99.6)	48.8	<0.001
≥ 4	71.4 (47.8-88.7)	80.9 (77.3-84.2)	13.2 (7.6-20.8)	98.6 (97.0-99.5)	52.4	<0.001
≥ 5	57.1 (34.0-78.2)	85.5 (82.2-88.5)	13.8 (7.3-22.9)	98.0 (96.3-99.1)	42.7	<0.001
≥ 6	38.1 (18.1-61.6)	88.2 (85.2-90.9)	11.6 (5.1-21.6)	97.2 (95.3-98.5)	26.3	0.002
≥ 7	33.3 (14.6-57.0)	90.0 (87.1-92.4)	11.9 (4.9-22.9)	97.1 (95.2-98.4)	23.3	0.004
≥ 8	33.3 (14.6-57.0)	90.4 (87.5-92.8)	12.3 (5.1-23.7)	97.1 (95.2-98.4)	23.7	0.004
≥ 9	28.6 (11.3-52.2)	90.9 (88.1-93.3)	11.3 (4.3-23.0)	96.9 (95.0-98.3)	19.5	0.011
≥ 10	28.6 (11.3-52.2)	91.3 (88.6-93.6)	11.8 (4.4-23.9)	96.9 (95.0-98.3)	19.9	0.009

AUC: 0.807 (95% CI: 0.723–0.892)

B. Considering all visits. *A single participant may contribute up to three observations if they completed questionnaires at multiple visits. (N = 2175; 30 events in 29 participants).*

Cut-off	SEN % (95% CI)	SPE % (95% CI)	PPV % (95% CI)	NPV % (95% CI)	Youden Index	*P*
≥ 1	63.3 (43.9-80.1)	73.8 (71.8-75.6)	3.3 (2.0-5.1)	99.3 (98.8-99.7)	37.1	<0.001
≥ 2	50.0 (31.3-68.7)	82.4 (80.7-84.0)	3.8 (2.2-6.2)	99.2 (98.6-99.5)	32.4	<0.001
≥ 3	43.3 (25.5-62.6)	87.6 (86.1-88.9)	4.6 (2.5-7.8)	99.1 (98.6-99.5)	30.9	<0.001
≥ 4	43.3 (25.5-62.6)	90.9 (89.6-92.0)	6.2 (3.4-10.4)	99.1 (98.6-99.5)	34.2	<0.001
≥ 5	30.0 (14.7-49.4)	92.6 (91.4-93.7)	5.4 (2.5-10.0)	99.0 (98.4-99.4)	22.6	<0.001
≥ 6	23.3 (9.9-42.3)	94.3 (93.2-95.2)	5.4 (2.2-10.8)	98.9 (98.3-99.3)	17.6	0.001
≥ 7	20.0 (7.7-38.6)	95.0 (94.0-95.9)	5.3 (2.0-11.2)	98.8 (98.3-99.3)	15.0	0.004
≥ 8	20.0 (7.7-38.6)	95.2 (94.2-96.0)	5.5 (2.0-11.5)	98.8 (98.3-99.3)	15.2	0.003
≥ 9	16.7 (5.6-34.7)	95.6 (94.6-96.4)	5.0 (1.6-11.3)	98.8 (98.2-99.2)	12.2	0.011
≥ 10	16.7 (5.6-34.7)	95.8 (94.8-96.6)	5.2 (1.7-11.7)	98.8 (98.2-99.2)	12.4	0.009

AUC: 0.708 (95% CI: 0.611–0.805)

Abbreviations: CRAI, condomless receptive anal intercourse; ROC, Receiver Operating Characteristic; SEN, Sensitivity; CI, Confidence Interval; SPE, Specificity; PPV, Positive Predictive Value; NPV, Negative Predictive Value; AUC, Area Under the Curve.

Table S7. Variables associated with HCV infections among 540 MSM with HIV who completed at least one App-based questionnaire, using generalized estimating equation (GEE) models to account for within-subject correlations over time.

Variable*	Univariate OR (95% CI)	P-value	Multivariate OR (95% CI)	P-value
Age	1.00 (0.96–1.04)	0.932	-	-
Born in Spain	0.94 (0.39–2.27)	0.891	-	-
History of prior HCV	3.48 (1.40–8.64)	0.007	1.64 (0.59–4.53)	0.341
CRAI	3.15 (1.05-9.45)	0.041	1.02 (0.32-3.27)	0.970
CRAI partners ≥ 4	6.80 (2.79-16.6)	<0.001	2.41 (0.82–7.07)	0.110
Fisting	8.00 (3.38-18.9)	<0.001	2.56 (0.92–7.13)	0.073
STI any	1.22 (0.41-3.65)	0.726	-	-
Syphilis	1.42 (0.33-6.11)	0.634	-	-
Gonorrhea	1.38 (0.32-5.98)	0.670	-	-
Chlamydia	1	-	-	-
Frequency of drug use	1.49 (1.20-1.86)	<0.001	1.02 (0.65-1.60)	0.942
Chem sex	7.25 (2.64-19.9)	<0.001	1.11 (0.15-8.14)	0.920
Methamphetamine use	7.04 (2.97-16.7)	<0.001	1.48 (0.51-4.28)	0.468
Mephedrone use	6.88 (2.76-17.1)	<0.001	1.05 (0.93-3.40)	0.939
GHB use	6.68 (2.81-15.9)	<0.001	1.60 (0.58-4.23)	0.364
Needle Sharing	1	-	-	-
Slam Sex	10.70 (4.56-24.9)	<0.001	3.50 (1.29–9.50)	0.014

Abbreviations: OR, odds ratio; CI, confidence interval; HCV, hepatitis C virus; CRAI, condomless receptive anal intercourse; STI, sexually transmitted infection; Chemsex, drug use during sex; GHB, gamma-hydroxybutyrate; Slam sex, intravenous drug use during sex.

*Variables related to sexual activity and drug use reflect behaviors within the two months preceding questionnaire completion. The threshold of ≥4 CRAI partners during this two-month period was determined using receiver operating characteristic (ROC) curves and the Youden test. STIs refer to any diagnosis of a sexually transmitted infection during the study period. The frequency of drug use was evaluated as an ordinal variable with the following categories: daily, weekly, biweekly, monthly, annually, or no drug use.

Table S8. Multivariate analysis of factors associated with HCV infections among MSM participants with or without HIV infection using Firth's penalized logistic regression with multiple imputation.

Variable	Multivariate OR (95% CI)	P-value
Age (per 10 years)	0.84 (0.55–1.29)	0.433
HIV infection	1.44 (0.56-3.71)	0.449
Born in Spain	1.27 (0.60–2.70)	0.530
CRAI	13.93 (0.81–238.28)	0.069
Chem sex	2.50 (1.00–6.23)	0.050
Slam sex	4.49 (1.90–10.60)	0.001
Prior HCV	2.74 (1.05–7.17)	0.040
Prior syphilis	2.12 (0.73–6.16)	0.169
Prior gonorrhea	1.19 (0.51–2.80)	0.692
Prior chlamydia	1.24 (0.53–2.88)	0.624

Abbreviations: OR, odds ratio; CI, confidence interval; HCV, hepatitis C virus; Chemsex, drug use during sex; Slam sex, intravenous drug use during sex.

Slam sex and prior HCV infection were significantly associated with HCV infection. CRAI showed a high odds ratio but with a wide confidence interval due to the absence of cases in the non-CLAI group. Chemsex presented a borderline association. Other factors, including age, country of birth, HIV status, and previous STIs, did not show significant associations with HCV infection.

Appendix I: Baseline and Follow-up Assessments for MSM with HIV (eCRF-Based)

Demographics and Medical History

Inclusion Criteria:

- Signed informed consent: Yes / No
- HIV infection status: Yes / No
- Men who have sex with men (MSM): Yes / No

Socio-demographic Data:

- Sex at birth: Male / Female
- Date of birth: __________
- Age: __________
- Country of birth: Spain: Yes / No (if no, specify country)

Race:

- White
- Black
- Other
- Not Available

Education Level:

- No primary education completed
- Primary education completed
- Secondary education completed
- University education completed
- Other

HIV Infection Status:

- CDC clinical category: A1, B1, C1, A2, B2, C2, A3, B3, C3
- Antiretroviral therapy (ART): Yes / No
- Current ART regimen (check all that apply): AZT, 3TC/FTC, TDF/TAF, ABC, EFV, NVP, ETR, RPV, DOR, ATV, DRV, LOP, DTG, RAL, BIC, EVG, LAI-CAB, LAI-RIL, FOST, Other (specify)

Previous Hepatitis C (HCV) Infection:

- Previous HCV infection: Yes / No
- Number of previous HCV infections: __________
- Previously treated for HCV: Yes / No
- Spontaneous clearance of previous infection: Yes / No
- Previous treatment regimen (if applicable): Peg-INF+RBV, IP+Peg-INF+RBV, SOF+RBV, SOF+SIM, SOF+DCL, 2/3D, ELB/GRA, GLE/PIB, SOF/LDV, SOF/VEL, SOF/VEL/VOX, Other (specify)
- Treatment duration (weeks): 6, 8, 12, 16, 24, 48
- Date of sustained virologic response (SVR): __________
- HCV genotype: 1a, 1b, 2, 3, 4, 5, 6, Unknown

Sexually Transmitted Infections (STIs) History:

- Syphilis: Yes / No
- Gonorrhea: Yes / No
- Chlamydia (non-LGV): Yes / No
- Lymphogranuloma venereum (LGV): Yes / No
- Hepatitis A: Yes / No
- Hepatitis B: Yes / No

Baseline and Follow-up Visit (6m and 12m) Assessments:

Clinical

- Visit completed: Yes / No (If No, specify reason)
- Date of visit: __________
- Incident infections since the last visit: Yes / No
  - HCV infection: Yes / No (Date: __________, Reinfection: Yes / No)
  - Syphilis: Yes / No (Date: __________, Type: primary, secondary, latent)
  - Chlamydia: Yes / No (Date: __________, Site: genital, anal, pharyngeal, LGV: Yes / No)
  - Gonorrhea: Yes / No (Date: __________)

Laboratory

- CD4+ cell count: _________ cells/mm³
- CD8+ cell count: _________ cells/mm³
- HIV viral load: Detectable / Undetectable (<50 copies/mL)
- HCV serology: Negative / Positive
- HCV RNA PCR: Positive / Negative
- Syphilis serology: Reactive / Non-reactive
- Chlamydia PCR: Positive / Negative
- Gonorrhea PCR: Positive / Negative

Incident HCV Infection Assessment:
- Diagnosis date: _________
- Reinfection: Yes / No
- Symptoms present: Yes / No (Specify symptoms)
- AST: _________
- ALT: _________
- HCV viral load: _________
- Genotype: 1a, 1b, 2, 3, 4, 5, 6, Unknown
- Treatment initiated: Yes / No (Specify regimen and duration: 6, 8, 12, 16, 24 weeks)
- Treatment response: Undetectable (SVR at week 12 or 24) / Detectable (treatment failure) / Ongoing (pending)

Study Completion:
- Reason for study termination: _________
- Date of completion: _________

Appendix II: App-Based Data on Sexual Practices, Drug Use, and Mental Health in MSM with HIV

Data on sexual behavior, substance use, and mental health were collected using structured questionnaires administered via REDCap. These questionnaires were provided directly by study investigators to participants living with HIV and were self-completed by each participant at baseline and subsequent study visits. Only individuals with confirmed HIV infection completed this module. REDCap was used both to distribute the surveys and to securely download the participant responses for analysis.

Var	Field label	Field attributes
3	Date	Text field, date (DD-MM-YYYY)
4	Have you used drugs in the last 2 months?	Radio: 0 = No, 1 = Yes
5	Tick the drugs you have used in the last 2 months (select all that apply)	Checkbox *(shown if variable 4 = 1)*1 Cocaine · 2 Cathinones · 3 Methamphetamines · 4 Ketamine · 5 GHB · 6 Ecstasy · 8 Marijuana · 9 Amphetamines · 7 Other
6	You indicated other drugs—please specify	Text field *(shown if variable 5 choice 7 = checked)*
7	In what context did you use these drugs?	Radio *(shown if variable 4 = 1)*: 0 Non-sexual · 1 Sexual · 2 Both
8	Route(s) of administration in the last 2 months (select all that apply)	Checkbox *(shown if variable 4 = 1)*0 Ingested · 1 Snorted · 2 Smoked · 3 Rectal · 4 Injected
9	Have you shared injection material?	Radio *(shown if variable 8 choice 4 = checked)*: 0 No · 1 Yes
10	How often do you use these drugs?	Radio *(shown if variable 4 = 1)*0 Daily · 1 Weekly · 2 Fortnightly · 3 Monthly · 4 Yearly
11	Have you had condom-less anal sex in the last 2 months?	Radio: 0 No · 1 Yes
12	If yes, indicate the role	Radio *(shown if variable 11 = 1)*1 Receptive · 2 Insertive · 3 Both
13	During condom-less anal sex in the last 2 months, with how many different partners?	Text field (integer) *(shown if variable 11 = 1)*
14	Have you practised "fisting"?	Radio: 0 No · 1 Yes
14a	HADS instructions	Descriptive text (no data captured)
15	I feel tense or nervous	Radio: 3 Almost all day · 2 Most of the day · 1 Sometimes · 0 Never
16	I still enjoy the things I used to	Radio: 0 Certainly, as before · 1 Not as much · 2 Only a little · 3 Not at all
17	I have a feeling that something awful is going to happen	Radio: 3 Yes, very much · 2 Yes, but not intense · 1 Yes, but not worrying · 0 Not at all
18	I can laugh and see the funny side of things	Radio: 0 As always · 1 A bit less · 2 Much less · 3 Not at all
19	My head is full of worries	Radio: 3 Almost all day · 2 Most of the day · 1 Sometimes · 0 Never
20	I feel happy	Radio: 3 Never · 2 Very seldom · 1 Sometimes · 0 Most of the day
21	I can sit still and relaxed	Radio: 0 Always · 1 Often · 2 Rarely · 3 Never
22	I feel slow and clumsy	Radio: 3 Most of the day · 2 Often · 1 Sometimes · 0 Never
23	I have an unpleasant "knots and tingling" feeling in my stomach	Radio: 0 Never · 1 Sometimes · 2 Often · 3 Very often
24	I have lost interest in my personal appearance	Radio: 3 Completely · 2 Neglect myself · 1 Maybe neglect · 0 As always
25	I feel restless, as if I cannot keep still	Radio: 3 Very much · 2 Quite a lot · 1 Not much · 0 Never
26	I look forward to things with pleasure	Radio: 0 As always · 1 A little less · 2 Much less · 3 Not at all
27	I suddenly feel panic or intense fear	Radio: 3 Very often · 2 Fairly often · 1 Rarely · 0 Never
28	I enjoy a good book, radio or TV programme	Radio: 0 Often · 1 Sometimes · 2 Rarely · 3 Almost never
29	ASRS-v1.1 instructions (last 6 months)	Descriptive text (no data captured)
30	How often do you struggle to finish the final details of a project?	Radio: 1 Never · 2 Rarely · 3 Sometimes · 4 Often · 5 Very often
31	How often do you have difficulty organising tasks?	Radio (same scale as 30)
32	How often do you forget appointments or obligations?	Radio (same scale as 30)

33	When a task requires a lot of thought, how often do you delay starting?	Radio (same scale as 30)
34	How often do you fidget with hands or feet when you have to sit for long?	Radio (same scale as 30)
35	How often do you feel over-active as if driven by a motor?	Radio (same scale as 30)
35a	Impulsivity questionnaire items instructions	Descriptive text (no data captured)
36	I plan my tasks carefully	Radio, matrix: 0 Rarely/never · 1 Occasionally · 3 Often · 4 Always/almost always
37	I do things without thinking	Radio, matrix (same scale as 36)
38	I hardly ever take things to heart	Radio, matrix (same scale as 36)
39	My thoughts can race	Radio, matrix (same scale as 36)
40	I plan my trips in advance	Radio, matrix (same scale as 36)
41	I am a self-controlled person	Radio, matrix (same scale as 36)
42	I concentrate easily	Radio, matrix (same scale as 36)
43	I save money regularly	Radio, matrix (same scale as 36)
44	I find it hard to stay still for long periods	Radio, matrix (same scale as 36)
45	I think things through carefully	Radio, matrix (same scale as 36)
46	I plan for steady employment	Radio, matrix (same scale as 36)
47	I say things without thinking	Radio, matrix (same scale as 36)
48	I enjoy thinking about complex problems	Radio, matrix (same scale as 36)
49	I change jobs frequently	Radio, matrix (same scale as 36)
50	I act impulsively	Radio, matrix (same scale as 36)
51	I get bored solving problems in my head	Radio, matrix (same scale as 36)
52	I visit doctor and dentist regularly	Radio, matrix (same scale as 36)
53	I do things as soon as they occur to me	Radio, matrix (same scale as 36)
54	I am a person who thinks without distraction	Radio, matrix (same scale as 36)
55	I move house often	Radio, matrix (same scale as 36)
56	I buy things impulsively	Radio, matrix (same scale as 36)
57	I finish what I start	Radio, matrix (same scale as 36)
58	I walk and move quickly	Radio, matrix (same scale as 36)
59	I solve problems by experimenting	Radio, matrix (same scale as 36)
60	I spend more than I earn (cash or credit)	Radio, matrix (same scale as 36)
61	I speak quickly	Radio, matrix (same scale as 36)
62	I have strange thoughts while thinking	Radio, matrix (same scale as 36)
63	I am more interested in the present than the future	Radio, matrix (same scale as 36)
64	I feel restless in classes or lectures	Radio, matrix (same scale as 36)
65	I plan for the future	Radio, matrix (same scale as 36)

Field type definitions: "Radio" refers to a single-choice field (i.e. only one response is allowed); "Checkbox" allows multiple selections; "Text" refers to open-entry fields, sometimes with validation rules (e.g. date, integer); "Matrix" indicates grouped Likert-type items displayed in tabular format; "Descriptive" denotes non-response fields used to provide instructions or context. "Var" indicates the variable number as listed in the REDCap codebook.

Abbreviations: HIV: Human Immunodeficiency Virus; STI: Sexually Transmitted Infection; LGV: Lymphogranuloma Venereum; GHB: Gamma-Hydroxybutyrate; GFR/eGFR: Glomerular Filtration Rate / Estimated Glomerular Filtration Rate; CD4: Cluster of Differentiation 4 (T-cell subset); RNA: Ribonucleic Acid; ASRS: Adult ADHD Self-Report Scale; HADS: Hospital Anxiety and Depression Scale; SMAQ: Simplified Medication Adherence Questionnaire.

Appendix III: Baseline and Follow-up Assessments for MSM without HIV on PrEP

Assessments followed the *HIV Pre-Exposure Prophylaxis (PrEP) Program Procedure for Adults and Adolescents, published by the Community of Madrid Regional Health Authority* (Vice-Consejería de Asistencia Sanitaria y Salud Pública, 25 May 2022). The procedure mandates:

Phase	Core components required by the regional procedure
Baseline visit (Week 0)	• Full medical history, sexual-behavior and substance-use assessment.<br>• Fourth-generation HIV ELISA (mandatory) ± plasma HIV-RNA if acute infection suspected.<br>• Comprehensive STI screen: *Neisseria gonorrhoeae*, *Chlamydia trachomatis* (including LGV), *Mycoplasma genitalium*, syphilis, herpes.<br>• Hepatitis panel: HAV, HBV (HBsAg, anti-HBc IgG/IgM, anti-HBs), HCV antibodies ± HCV RNA.<br>• Safety panel: complete blood count, electrolytes, serum creatinine, estimated glomerular filtration rate (eGFR), serum phosphate, urinalysis (dipstick protein, glucose, sediment) and protein/creatinine ratio.<br>• Clinical counselling on adherence and risk-reduction.
Quarterly visits	• Repeat adherence, safety and risk assessments.<br>• HIV test (fourth-generation ELISA or qualitative HIV-RNA).<br>• Full STI screen.<br>• Renal function (serum creatinine, eGFR, urinalysis, protein/creatinine ratio) every 3 months<br>• Continue or discontinue PrEP according to risk profile, toxicity or patient preference.

Abbreviations: PrEP: Pre-Exposure Prophylaxis; HIV: Human Immunodeficiency Virus; STI: Sexually Transmitted Infection; LGV: Lymphogranuloma Venereum; HAV: Hepatitis A Virus; HBV: Hepatitis B Virus; HBsAg: Hepatitis B Surface Antigen; anti-HBc: Antibodies to Hepatitis B Core Antigen; anti-HBs: Antibodies to Hepatitis B Surface Antigen; HCV: Hepatitis C Virus; RNA: Ribonucleic Acid; eGFR: Estimated Glomerular Filtration Rate; ELISA: Enzyme-Linked Immunosorbent Assay

Participant data were collected using a mandatory standardized form issued by the Regional Ministry of Health (The RUA registry). This registry form included the following variables:

Var	Field label (EN)	Var	Field label (EN)
1	Participant ID	28	Mycoplasma (test result)
2	Country of birth	29	Mycoplasma site of infection
3	Visit type (Baseline / Follow-up)	30	Syphilis (test result)
4	Date of cohort inclusion	31	Syphilis stage
5	Age at visit	32	Herpes (test result)
6	Percentage of anal sex acts with condom	33	Herpes site of infection
7	Number of sexual partners	34	Hepatitis A (test result)
8	Drug use in the past 2 months (Yes/No)	35	Hepatitis B (test result)
9	Use of intravenous drugs (injectable stimulants)	36	Hepatitis C (antibodies test result)
10	Use of drugs in a sexual context (chemsex)	37	HBV immunity status
11	Number of sexual encounters	38	HBV infection status
12	HIV test performed	39	HCV infection status (HCV RNA)
13	HIV test result	40	HBV DNA status
14	Western blot (confirmatory test)	41	Vaccination record
15	HIV viral load	42	Renal impairment
16	CD4 cell count	43	Serum creatinine
17	Suspected route of HIV acquisition	44	Estimated glomerular filtration rate (eGFR)
18	Main reason for initiating PrEP	45	Urine protein (dipstick)
19	Eligibility criteria for PrEP	46	Protein-to-creatinine ratio

20	Engagement in sex work	47	Serum phosphate
21	Previous use of PrEP	48	Urinary glucose (dipstick)
22	STD screening performed	49	Urinary sediment
23	Chlamydia (test result)	50	Notes on urinary sediment
24	Chlamydia site of infection	51	Pregnancy test result
25	Lymphogranuloma venereum	52	PrEP discontinued (temporary or permanent) (Yes/No)
26	Gonorrhea (test result)	53	Reason for discontinuation
27	Gonorrhea site of infection	54	Other reason for discontinuation

Abbreviations: PrEP: Pre-Exposure Prophylaxis; HIV: Human Immunodeficiency Virus; STD: Sexually Transmitted Disease; STI: Sexually Transmitted Infection; HBV: Hepatitis B Virus; HCV: Hepatitis C Virus; RNA: Ribonucleic Acid; DNA: Deoxyribonucleic Acid; eGFR: Estimated Glomerular Filtration Rate; CD4: Cluster of Differentiation 4 (T lymphocyte subset).

Appendix IV: Detailed Statistical Analysis

Descriptive Analysis:

- Descriptive statistics summarized all study variables.
- Normality of continuous variables was assessed using frequency histograms and the Shapiro-Wilk test.
- Baseline prevalence and incidence rates (per 100 person-years, PY) of active hepatitis C virus (HCV) infection during follow-up were estimated with corresponding 95% confidence intervals (CIs).
- Participants with active HCV infection at baseline were excluded from incidence analyses.
- All analyses were stratified by prior HCV infection history and HIV status.

Incidence Analysis:

- Prevalence ratios and IRRs with 95% CIs were calculated using Poisson regression, comparing participants with and without prior HCV infection.
- Incident infections in participants without prior HCV infection history and negative baseline HCV RNA were classified as primary infections. All other incident infections were classified as reinfections. Incidence rates were calculated from baseline until the date of confirmed infection.
- Within-study reinfections were separately analyzed, defined as a new positive HCV RNA result after a previous negative test in participants with active HCV infection detected at any point during the study. In this case, incidence rates were calculated from the first negative RNA test after initial positivity and was censored at the next positive test, the last available RNA result, or end of follow-up.

Multivariable Analysis:

- Logistic regression identified factors associated with HCV infection events.
- The primary analysis focused on MSM with HIV due to detailed self-reported data collected via comprehensive questionnaires on sexual behaviors and drug use administered through a mobile application.
- HCV infections lasting less than 12 months (per NEAT-ID Consensus Panel criteria) were combined into one category, assuming similar population characteristics and transmission dynamics. Within-study reinfections were excluded from this analysis.

Independent Variables:

- Independent variables selected based on clinical relevance included:
  - Age (assessed at baseline)
  - Born in Spain (assessed at baseline)
  - Prior HCV infection (assessed at baseline)
  - Condomless receptive anal intercourse (CRAI) in the two months preceding questionnaire completion
  - Number of CRAI partners (≥4 partners, threshold determined by receiver operating characteristic (ROC) curves and the Youden test)
  - Fisting within the previous two months preceding questionnaire completion.
  - Frequency of drug use (evaluated ordinally: daily, weekly, biweekly, monthly, annually, or no drug use)
  - Chemsex preceding in the two months preceding questionnaire completion
  - Methamphetamine use in the two months preceding questionnaire completion
  - Mephedrone use in the two months preceding questionnaire completion
  - Gamma-Hydroxybutyrate (GHB) use in the two months preceding questionnaire completion
  - Needle sharing
  - Slamsex in the two months preceding questionnaire completion
  - Any STI diagnosis during the study period, specifically: Syphilis, Gonorrhea, and Chlamydia
- Data from baseline, month 6, and month 12 questionnaires were incorporated.
- Drug use and sexual behaviors were analyzed categorically and ordinally based on frequency. The highest reported category or frequency during any study visit was utilized.
- Variables with a P-value < 0.05 in univariate analysis were included in the final multivariable model.

Validation of Findings:

- Temporal distributions of key risk behaviors were compared across baseline, month 6, and month 12 visits using chi-squared tests (categorical variables) and independent-group comparisons (continuous variables).
- Within-subject correlations were evaluated using Stuart-Maxwell tests for symmetry and marginal homogeneity.

Sensitivity Analyses:

- Sensitivity analyses among MSM with HIV employed generalized estimating equations (GEE) models, assuming an exchangeable correlation structure.
- Additional sensitivity analyses differentiated primary infections from reinfections using logistic regression and GEE models.
- Another sensitivity analysis included all participants regardless of HIV status, using Firth's penalized logistic regression to minimize bias from low event frequencies, examining associations with common baseline variables.

Missing Data:

- Missing data were addressed using multiple imputation by chained equations.
- Little's MCAR test confirmed that the missing data did not follow a completely random pattern.

Statistical Software and Guidelines:

- Statistical analyses used SPSS version 25 and STATA 18.0 (STATA Corp., College Station, TX, USA).
- All statistical tests were two-sided.
- Reporting adhered to STROBE guidelines for transparency and accuracy.

Appendix V. GeSIDA 12121-RIS EPICLIN 08_2021 ATHENS Study Group

Hospital General Universitario Gregorio Marañón: L Pérez-Latorre, T Aldámiz-Echevarría, C Díez, F Tejerina, C Fanciulli, CA Fredes, P Catalán, M Ramírez-Schacke, I Gutiérrez, JC López, JM Bellón, J Berenguer.

Hospital Universitario La Paz: MM Arcos Rueda, JR Arribas López, V Arribas Santos, JI Bernardino de la Serna, C Busca Arenzana, JM Castro Álvarez, A Delgado Nieto, A de Gea Grela, R de Miguel Buckley, R Micán Rivera, ML Martín-Carbonero, R Montejano Sánchez, ML Montes Ramírez, CM Oñoro López, L Ramos Ruperto, J Cano Smith, E Valencia Ortega, and J González-García.

Hospital Universitario Infanta Leonor: J Valencia, G Cuevas, M Matarranz, J Troya, L Laguna, P Torres, P Parbole, S Manzano, S Estévez Alonso, D Brown, E Fernández Vidal, JD Arroyo-Moreno, C Culebras-Villalva, C Rodríguez-Lescure, VE Gonzalez-Gutierrez, E Peñacoba Páramo, J Torres-Macho, P Ryan.

Centro Sanitario Sandoval: M Vera, C Rodríguez, O Ayerdi, J Del Romero.

Hospital Universitario de La Princesa: L García-Fraile, A Bautista, C Sáez, A Barrios, A Gutiérrez-Liarte, M Ciudad, A Casen, I De Los Santos.

Hospital Universitario 12 de Octubre: A Pinto, M De Lagarde, L Bermejo-Plaza.

Hospital Universitario Ramón y Cajal: MJ Vivancos, S Del Campo, A Moreno-Zamora, MJ Pérez-Elías, C Quereda, JL Casado, S. Moreno.

Hospital Clínico de San Carlos: E Orviz, R Homen, MJ Núñez, N Cabello, J Pérez-Somarriba, J Rodríguez-Añover, V Estrada.

Hospital Universitario Fundación Jiménez Díaz: B Álvarez, L Prieto, M Górgolas, A Cabello.

Hospital Universitario Príncipe de Asturias: C Hernández, M Novella, J Sanz.

Hospital Universitario Infanta Sofía: P Ruiz-Seco, P González-Ruano, I Suarez-García, JM Maza

Hospital Universitario Severo Ochoa: R Torres, M Cervero.

Subdirección General Farmacia Productos Sanitarios-SERMAS: B López-Centeno, A Gil-Martin, D Alioto.

Centro Nacional de Epidemiología-ISCIII: M Rava, R Izquierdo, T Gómez, C Marco, I Jarrín.

Centro Nacional de Microbiología-ISCIII: I Martínez, D Sepúlveda, A Virseda, R Amigot, MJ Muñoz, S Resino.

Fundación SEIMC/GeSIDA: B Brazal, H Esteban, M De Miguel.
